# Unraveling the invasiveness of *Prosopis juliflora* (*Neltuma juliflora*): seed traits and ecological implications

**DOI:** 10.3389/fpls.2025.1721722

**Published:** 2026-01-27

**Authors:** Kamel Chibani, Mengjie Fan, Hamada E. Ali, Laya Al-Kharusi

**Affiliations:** 1 Department of Biology, College of Science, Sultan Qaboos University, Muscat, Oman; 2 School of Life Sciences, University of Essex, Colchester, United Kingdom

**Keywords:** climate change, germination, invasion, Neltuma, phenotypic plasticity, Prosopis, seeds, stress tolerance

## Abstract

*Prosopis juliflora* (*Neltuma juliflora*) is a globally invasive tree species threatening arid ecosystems. Its invasion success is driven by specific seed traits that function as an adaptive bet-hedging strategy. The impermeable seed coat enforces physical dormancy and enables the formation of a persistent soil seed bank that buffers against environmental stochasticity (population insurance). Conversely, rapid germination allows the species to exploit short-lived moisture pulses and outcompete native vegetation. Livestock-mediated endozoochory further facilitates directed dispersal by depositing scarified seeds in favorable microsites. This mini-review synthesizes current knowledge on these anatomical and physiological mechanisms and examines how they interact with climate change variables, specifically rising temperatures and altered precipitation on intensifying invasion dynamics. Finally, we discuss integrated management strategies targeting seed bank depletion and dispersal pathways.

## 1 Introduction

*Prosopis juliflora*, commonly known as the mesquite plant, is an invasive tree that belongs to the Fabaceae family and the Caesalpinioideae subfamily (Mimosoid clade) (Hussain et al., 2020). The Prosopis genus comprises 56 species that differ primarily in armature characteristics (spine morphology), fruit architecture, and geographic distribution (Hughes et al., 2022). Among these species, four are recognized for their exceptional invasiveness: *P.juliflora* across East Africa, India, Pakistan, and the Arabian Peninsula; and *Prosopis pallida* in regions of Africa and Australia; *Prosopis glandulosa* in southern Africa and Australia; *Prosopis velutina* in parts of Australia (van Klinken and Campbell, 2001; Castillo et al., 2020; Patzelt et al., 2022).

*P.juliflora* is considered the most invasive species within the Prosopis genus because of its rapid canopy expansion, severe suppression of native vegetation, well-known allelopathic effects (El-Keblawy and Al-Rawai, 2006; Tsombou et al., 2022; Abbas et al., 2023), and unique tetraploid status (2n=4x=56) that distinguishes it from diploid congeners and provides enhanced stress tolerance (Trenchard et al., 2008; Castillo et al., 2020). Recent molecular phylogenomic evidence and morphological analyses have led to the taxonomic reclassification of *P.juliflora* from Prosopis genus due to non-monophyletic ancestry and their distinct evolutionary lineage. This revision split Prosopis (sensu lato) into three genera: Prosopis sensu stricto, Neltuma, and Strombocarpa, and *Prosopis juliflora* is now treated as *Neltuma juliflora* (Hughes et al., 2022). Distinct morphological differences, particularly in pod structures, seed morphology, and ​floral traits, as well as molecular evidence, have led to the reclassification of *Prosopis juliflora* as *Neltuma juliflora* (Hughes et al., 2022).

*P.juliflora* is listed among the top 100 worst invasive species globally, with severe ecological and socioeconomic impacts (Lowe et al., 2000; Paliwal et al., 2024). Native to Mexico, central America, and northern south America, particularly Peru, this species has been extensively introduced in Africa, Asia, Australia, and Middle East regions (Shackleton et al., 2015), currently covering approximately 500,000 km² globally, with expansion rates of 640 hectares per year in Kenya and 31,127 hectares per year in Ethiopia (Mbaabu et al., 2019; Sintayehu et al., 2020). It is considered genetically different from other species in the genus because of its unique ploidy level (Harris et al., 2003; Trenchard et al., 2008). While most Prosopis species are diploids with limited genetic variation, *P.juliflora* is tetraploid (2n=4x=56), as demonstrated by cytogenetic and foliar morphological analyses (Trenchard et al., 2008). This distinctive ploidy level may contribute to its invasiveness and adaptive capacity under various environmental stresses, which facilitates its high distribution rate worldwide (Castillo et al., 2020), providing genomic buffering against environmental variation and enhanced stress-responsive gene expression (Kong et al., 2023).

Originally introduced primarily for desertification control owing to its exceptional ability to survive in arid and semi-arid regions, particularly in saline and sandy soils, *P.juliflora* effectively controls soil erosion and contributes to landscape restoration (Ghazanfar, 1997; Al-Rawahy et al., 2003; Vargas-Lomelín et al., 2022). Despite its ecological benefits in certain contexts, *P.juliflora* serves as a reservoir of allergens that pose threats to human and animal health, with its allergenic pollen provoking severe respiratory problems (Hussain et al., 2020). *P.juliflora* now competes vigorously with native vegetation and poses a significant threat to the biodiversity of various plant communities, with soil seed bank diversity declining by 19.2% in invaded areas and native species richness reduced by 32-78% compared to uninvaded sites (Kaur et al., 2012; Shiferaw et al., 2019).

Moreover, the presence of *P.juliflora* can intensify the invasional meltdown phenomenon by altering ecosystem dynamics through plant-soil feedback, which has positive effects on subsequent invasives (+24.2% biomass increase) while inhibiting native species (-27.7% biomass decrease), thereby facilitating the spread of other invasive species (Simberloff and Von Holle, 1999; Ali et al., 2024). Control efforts are being made to control its invasiveness in the ecosystem such as the mechanical removal, use of herbicides including new formulations like Sendero^®^ (aminopyralid + clopyralid), biological control agents, and ecological restoration strategies (Ali et al., 2024; Eschen et al., 2023). Restoration of native species following *P.juliflora* mechanical removal has been adopted in several countries; however, seed dispersal presents significant management challenges (Tewari et al., 2001; Shiferaw et al., 2004). The prolific seed production (60,000-100,000 seeds per mature tree annually) and dispersal mechanisms make *P.juliflora* propagation control difficult, as mechanical clearing often leads to rapid reinvasion from persistent seed banks that remain viable for 40+ years (Miranda et al., 2011; Nascimento et al., 2020; Renzi et al., 2024).

Seeds play a major role in the life cycle of this species, with multiple factors influencing its invasiveness (Nigatu et al., 2019; Nascimento et al., 2020). These factors include exceptional seed production rates, rapid germination capacity (within 6 hours at optimal temperature of 35°C), diverse seed dispersal mechanisms, adaptability across a wide range of habitats with 200–1400 mm of annual rainfall, and remarkable stress tolerance enabling germination at water potentials down to -1.5 MPa (Haregeweyn et al., 2013; El-Keblawy and Al-Rawai, 2005; Hameed et al., 2021; Rajak et al., 2024). Despite its specific ploidy level, physiological characteristics, and ecological adaptations, the invasiveness of *P.juliflora* is primarily attributed to the specific physiological traits of its seeds (Baskin and Baskin, 1996; Miranda et al., 2011). The impact of seed physiology on *P.juliflora* invasiveness remains inadequately studied and discussed, particularly in the context of climate change which is projected to expand suitable habitat by 55-80% by 2070 through temperature increases and altered precipitation patterns (Sintayehu et al., 2020; Ahmad et al., 2019).

In this mini-review, we present (i) an overview of the current knowledge regarding the anatomical and physiological traits of *P.juliflora* that enhance seed germination, dormancy breaking, and environmental stress tolerance, incorporating recent advances in understanding phenotypic plasticity (Tilahun et al., 2025) and climate responses (Rajak et al., 2024), with a discussion of the potential applications of growth hormones and regulators to reduce germination and limit seed-based propagation, and (ii) a discussion of how seed traits contribute to the establishment of *P.juliflora* across diverse habitats under current and future climate scenarios and the challenges they pose for ecological control strategies.

## 2 Seeds traits and germination dynamics enabling invasive potential

**Figure 1:**
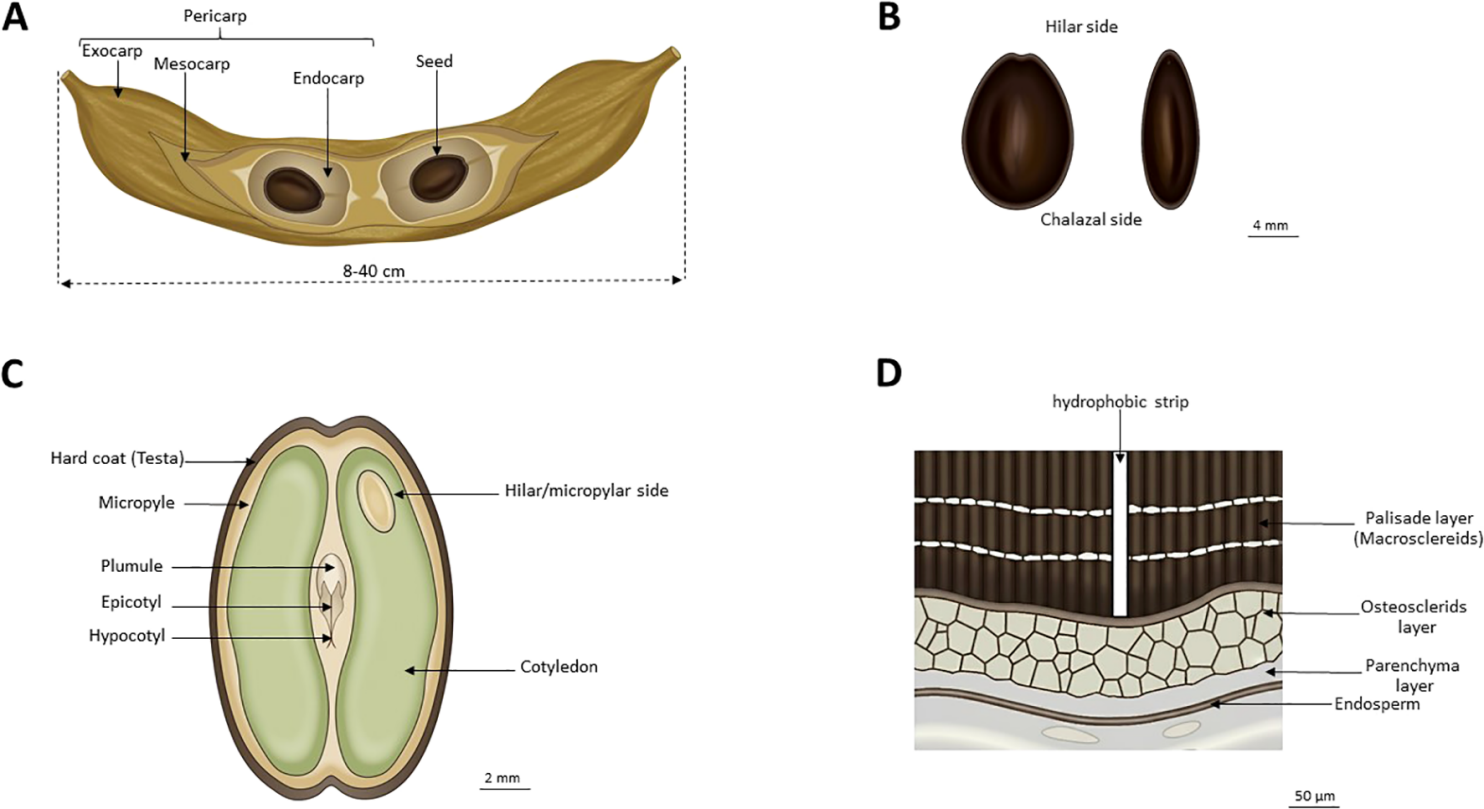
Morphology and anatomical features of *Prosopis juliflora* pod (fruit) and seed. **(A)** Whole pod (fruit); **(B)** External morphology of the seeds; **(C)** Longitudinal section of the seed; **(D)** Seed coat cross section.

The seeds of *P.juliflora* contribute significantly to its invasive capacity through multiple morphological and physiological adaptations, enhanced under elevated CO_2_ conditions projected for future climates. Seeds are enclosed in indehiscent yellow pods lacking internal septa, facilitating effective dispersal and environmental persistence (Marangoni and Alli, 1988). Pod dimensions vary from 10–20 cm in length and 1–2 cm in width, containing 10–30 seeds embedded in sweet (Pasiecznik et al., 2001). Each tree produces 60,000-100,000 seeds annually during fruiting seasons that span 3–4 months (**Figure 1A**).

Seeds are oval to elliptical, measuring 6.8-7.5 mm in length, 5.0-5.5 mm in width, and 2–3 mm in thickness, with mass ranging from 24–67 mg depending on environmental conditions and maternal effects (Pasiecznik et al., 2001; Renzi et al., 2024). The color ranges from light to dark brown, and it has a smooth, hard surface texture that resists mechanical damage. Recent work by Tilahun et al. (2025) documented significant genetic variation in seed mass (χ²=361.05, p<0.001) across Ethiopian populations, with seed mass varying 2.6-fold (7–18 g per tree) depending on habitat conditions, demonstrating both genetic and plastic components to this trait (**Figure 1B**).

The hard seed coat (testa) comprises a waxy cuticle and a palisade layer of lignified macrosclereids that form a water-impermeable barrier (**Figures 1C, D**) (Serrato-Valenti et al., 1990; Venier et al., 2012). Functionally, this barrier enforces physical dormancy, preventing germination during minor rainfall events that are insufficient for establishment. This mechanism supports the formation of persistent soil seed banks, with viability exceeding 80% after 10 years (Sawal et al., 2004; Renzi et al., 2024). Consequently, the seed bank acts as a temporal buffer, allowing populations to survive prolonged drought and rapidly recolonize following disturbance.

The endosperm, rich in nutrients, supports rapid germination and seedling establishment once dormancy breaks. Seeds contain 31.4-35.2% protein, 3.8-4.5% fat, and 56.2-59.3% carbohydrates (Marangoni and Alli, 1988). The galactomannan content, comprising 28-30% of the endosperm mass, serves dual functions as an energy reserve and water-binding agent, maintaining cellular hydration during germination under water stress conditions (Rincón et al., 2014). This hydrocolloid property is particularly advantageous during establishment in arid environments where water availability fluctuates.

The embryo comprises two large cotyledons containing storage proteins and lipids, a short hypocotyl, and a well-developed radicle positioned to emerge through the micropyle upon germination (**Figure 1C**) (Meyer et al., 1971). The embryonic axis shows no morphological dormancy, being fully developed at seed maturity. Histochemical analyses reveal concentrated protein bodies and lipid droplets in cotyledonary cells, providing immediate energy upon germination activation (Venier et al., 2012).

### 2.1 Germination dynamics and environmental responses

Germination in *P.juliflora* is highly plastic and strongly shaped by environmental cues. Optimal germination occurs at temperatures between 30-35°C, and the species shows the fastest germination among Prosopis, with radicle protrusion within 6 hours at 35°C (El-Keblawy and Al-Rawai, 2005). Mechanistically, this rapid germination velocity provides a competitive advantage over slower recruiting native species, allowing *P.juliflora* to preemptively capture soil moisture during ephemeral rainfall pulses. Under climate warming scenarios, these favorable temperature windows may expand germination periods by 30–45 days annually in many invaded regions (Sintayehu et al., 2020).

The species demonstrates exceptional tolerance to water stress, germinating at water potentials (Ψw) as -1.5 MPa under controlled laboratory conditions (El-Keblawy and Al-Rawai, 2005; Hameed et al., 2021). This contrast suggests ion regulation rather than simple osmotic adjustment. Stress tolerance also varies by seed origin: populations from saline habitats show 15-20% higher germination under severe stress, indicating transgenerational (Hameed et al., 2021). Light requirements are minimal. Seeds germinate equally well in continuous light or darkness at optimal conditions, but darkness enhances germination by 10-15% once the Ψw drops below -0.6 MPa (El-Keblawy and Al-Rawai, 2006), reflecting a preference for shaded microsites that reduce desiccation risk. Salinity tolerance is exceptional, with 40-50% germination maintained at 500 mM NaCl (El-Keblawy and Al-Rawai, 2005). Seeds demonstrate halophytic characteristics and remain viable after prolonged exposure and germinate rapidly once transferred to fresh water, demonstrating reversible dormancy under saline stress. Rainfall manipulation experiments reveal bidirectional plasticity. Low rainfall conditions (500 mm), promote faster germination, whereas high rainfall (1400 mm), delays germination delayed, but increase seedling vigor by 78%, demonstrating adaptive bet-hedging across rainfall gradients (Rajak et al., 2024).

### 2.2 Physical and physiological dormancy mechanisms

Physical dormancy in *P.juliflora* is imposed by a water-impermeable seed coat that blocks imbibition through a sealed hilum-micropylar region (Serrato-Valenti et al., 1990). This barrier forms through lignin and suberin deposition in palisade cells during seed maturation. Physical dormancy is released only when the testa is mechanically, chemically, thermally, or biologically disrupted. Mechanical scarification (abrasion or cutting) opens the coat, while concentrated sulfuric acid (98% for 10–20 minutes) effectively degrades the cuticle (Robles and Castro, 2002; Miranda et al., 2011; Smýkal et al., 2014). Dry heat (80-100°C) or boiling water (1–5 minutes) causes seed coat cracking (Villagra, 1997). Biological scarification such gut passage, especially 24–96 hours in cattle, provides efficient natural scarification (Kebede and Coppock, 2015). Fresh seeds show only 5-10% germination, and although aging weakens dormancy slightly, impermeability can persist for decades (Sawal et al., 2004). Dry storage maintains dormancy, whereas fluctuating humidity promotes microcracking of the testa. Dormancy depth varies among populations: seeds from thermally variable, unpredictable environments show deeper dormancy and 30-72% viability after 3 years, while seeds from stable habitats exhibit shallow dormancy and 0-10% viability (Tran and Cavanagh, 1984; Renzi et al., 2024). Unlike many legumes, *P.juliflora* lacks combinational dormancy, once the coat is broken, seeds either germinate immediately or lose viability within weeks (Miranda et al., 2011; Zare et al., 2011), making soil disturbance a strong promoter of invasion. This ‘all-or-nothing’ response to scarification means that physical disturbances such as livestock trampling or flash floods act as immediate triggers for mass recruitment.

## 3 Ecological implications and impact on invaded ecosystems

The invasion success of *P.juliflora* invasion results from strong interactions between seed traits and ecosystem processes that are increasingly mediated by climate change and human activities. These interactions create cascading effects that transform ecosystem structures and functions across multiple scales.

### 3.1 Dispersal mechanisms and spatial spread dynamics

*P.juliflora* dispersal is dominated by livestock-mediated dispersal. In pastoral ecosystems of northeastern Ethiopia, cattle mediated endozoochory was found to account for 92.9% of seed spread (Kebede and Coppock, 2015; Shiferaw et al., 2019). Cattle consume 2–5 kg of pods daily, with gut passage providing optimal scarification, and germination increases from <10% in intact seeds to 47-73% after cattle digestion (Nascimento et al., 2020). This constitutes a directed dispersal mechanism, with cattle scarifying seeds via gut passage and depositing them in nutrient rich dung, creating favorable microsites for establishment. This livestock dependence generates predictable spread along grazing routes, water points, and corrals, with invasions expanding at 640 ha/year in pastoral landscapes such as those in Kenya (Mbaabu et al., 2019). Secondary dispersal by floods extends spread into riparian zones, as seeds remain buoyant and viable for 30 days (Shiferaw et al., 2004). Human activities including the transport of pods, movement of hay, and machinery create a long-distance dispersal event that connect distant populations (Nascimento et al., 2020).

### 3.2 Seed bank dynamics and persistence

​ Large and persistent soil seed banks reinforce invasion. Densities can reach 50,578 seeds/m², with two-thirds concentrated in the litter layer and the remainder at 0–9 cm depth (Shiferaw et al., 2019). Burial enhances longevity: seeds at 10 cm depth maintain 80% viability after 3 years compared to 30% for surface seeds (Renzi et al., 2024). This depth-dependent persistence creates a vertical gradient of invasion potential, with deep seeds providing long-term population insurance while surface seeds enable rapid response to favorable conditions. Seed survival is shaped by moisture, temperature fluctuations (T>40°C), and soil chemistry (alkaline soils with pH 8-9), which promote persistence, while humidity and acidity accelerate decay. As invasions mature, allelopathy and prolific seed input result in near-monospecific seed banks, reducing native seed representation and slowing ecosystem recovery (Abbas et al., 2023). Models predict that seed banks may require >50 years to collapse even after complete removal of adult trees (Shiferaw et al., 2021).

### 3.3 Phenotypic plasticity and adaptation

High phenotypic plasticity strengthens invasion across environmental gradients. Seed mass, number and maturation vary widely among populations (Tilahun et al., 2025). Drought during seed development reduces individual seed mass by 30-40% but increases seed number by up to 25%, maintaining output. Under high nutrient availability, both seed size and number increase. Selection pressures differ across land-use types, with strong selection for early flowering and deeper dormancy in heavily grazed areas (Tilahun et al., 2025). Protected areas show relaxed selection (gradient=0.08), maintaining higher trait variation. Experimental rainfall manipulation demonstrates adaptive shifts: drought (500 mm annual rainfall), increase seed coat thickness and dormancy, whereas high rainfall (1400 mm) reduces dormancy and promote rapid germination (Rajak et al., 2024). Transgenerational plasticity further enhances stress tolerance, as seeds from drought-stressed mothers germinate 15-20% more under -1.5 MPa osmotic stress (Hameed et al., 2021).

### 3.4 Allelopathic interactions

Allelopathy suppresses native germination and strengthens dominance. Leachates containing phenolic alkaloids (juliflorine, julifloricine, and julifloridine), syringin, and L-tryptophan inhibit germination of co-occurring species (Nakano et al., 2003). Small-seeded species show >80% germination reduction. Autotoxicity occurs under deep leaf litter (8 cm), which reduces conspecific germination through combined physical and chemical mechanisms (Abbas et al., 2023). Soil conditioning studies demonstrate positive feedback for *P.juliflora* growth and negative effects on native species (Ali et al., 2024).

### 3.5 Ecosystem-level impacts

Invasion reduces seed bank diversity by 19.2% and native species richness by more than half (Shiferaw et al., 2019). Seed dispersal networks shift as frugivores preferentially consume *P.juliflora* pods, reducing dispersal of native plants (Kebede and Coppock, 2015). Hydrological changes further favor *P.juliflora*: high transpiration rates (3.1-3.3 billion m³/year consumption in Ethiopia’s Afar region) deplete water, creating drier microsites that disadvantage native seeds (Dzikiti et al., 2013). However, models projecting future warming scenarios suggest a potential 55–80% expansion in highly suitable habitat by 2070 (Haregeweyn et al., 2013; Sintayehu et al., 2020). These projections indicate that rising temperatures may release *P.juliflora* from current thermal constraints, allowing it to colonize higher latitudes or altitudes.

## 4 Discussion and future perspectives

### 4.1 Integration of seed traits with climate change projections

The invasion success of *P.juliflora* emerges from synergistic interactions between seed traits and environmental drivers, creating self-reinforcing cycles that resist conventional management. Climate change amplifies these mechanisms, making future invasions increasingly difficult to control. Rising temperatures favor germination by increasing the frequency of optimal temperature conditions (30-35°C), and by extending growing seasons Sintayehu et al. (2020). Elevated atmospheric CO_2_ (650–700 ppm) enhances seedling survival by 21-35% through improved water use efficiency and deeper rooting, giving *P.juliflora* a strong advantage during early establishment (Polley et al., 2002). Changes in precipitation produce region-specific effects: increased drought favors this species due its ability to germinate under low water potential, while intense rainfall events create windows of high soil moisture and enhance flood-mediated seed dispersal. The interaction between temperature and precipitation constrains native species, while *P.juliflora*, with it is extended fruiting period and persistent seed banks buffers against temporal variability.

### 4.2 Management implications, research priorities and future directions

Management strategies often fail because they address symptoms rather than underlying mechanisms that enable invasion. Clearing or herbicide applications kill standing trees but cannot address persistent seed banks. Effective control requires integrated approaches that reduce seed dispersal, and deplete seed reserves. Removing flowers or young pods prevents replenishment of the seed bank, while temporary livestock exclusion during peak fruiting can drastically reduce dispersal. Seed bank depletion can be promoted by controlling irrigation to induce synchronized germination followed by removal of seedlings, or by soil solarization to kill shallow seeds. Restoration of native plant communities after removal requires active reseeding and temporary exclusion of grazing (Linders et al., 2020).

Significant knowledge gaps remain. Genomic and molecular studies are needed to clarify mechanisms underlying dormancy, germination, plasticity, and transgenerational responses. CRISPR gene editing could create male-sterile lines that prevent seed production while maintaining trees for erosion control where complete removal proves impractical. RNA interference targeting seed-specific genes might prevent viable seed formation. The role of seed-associated microbiomes, long-term evolutionary dynamics under management pressure, and early detection technologies also represent research frontiers. Environmental DNA from soil or water could detect *P.juliflora* propagules before visible establishment. Hyperspectral remote sensing can distinguish *P.juliflora* from native vegetation based on unique spectral signatures. Machine learning algorithms trained on invasion patterns could predict spread trajectories. Citizen science networks using smartphone apps for identification and reporting could provide landscape-scale surveillance. Integration of these approaches with species distribution models would help prioritize areas for monitoring and preemptive management.

## 5 Conclusions

*Prosopis juliflora* exemplifies how seed traits can drive successful plant invasions through complex mechanisms operating across multiple scales. The combination of physical dormancy enabling decades-long persistence, exceptional stress tolerance allowing germination under extreme conditions, rapid germination when conditions become favorable, and efficient dispersal primarily through livestock (92.9% of spread), creates a formidable invasion syndrome. These traits, amplified by remarkable phenotypic plasticity documented across environmental gradients (Tilahun et al., 2025; Rajak et al., 2024) and enhanced by climate change projections showing 55-80% habitat expansion by 2070 (Sintayehu et al., 2020), position this species to become increasingly problematic globally.

The recent recognition that *P.juliflora* is the only tetraploid (2n=4x=56) among formerly recognized Prosopis species provides a genomic explanation for its superior invasiveness compared to diploid congeners. This polyploidy likely confers enhanced stress tolerance through gene dosage effects, buffering against environmental variation, and increased genetic variation for rapid adaptation.

Understanding seed physiology and dormancy regulation mechanisms in the context of global environmental change is essential for developing successful control strategies and effective ecological management approaches. The persistent seed banks, transgenerational plasticity, and rapid evolutionary potential of *P.juliflora* necessitate long-term, integrated management strategies that simultaneously target seed production, dispersal, germination, and establishment while actively restoring native communities. Climate change will likely exacerbate invasion risks through multiple pathways, requiring adaptive management frameworks that explicitly incorporate environmental uncertainty.
